# Making it to the Academic Path in a Tracked Education System: The Interplay of Individual Agency and Social Origin in Early Educational Transitions

**DOI:** 10.1007/s10964-023-01846-y

**Published:** 2023-09-02

**Authors:** Francesca Mele, Marlis Buchmann, Kaspar Burger

**Affiliations:** 1 Jacobs Center for Productive Youth Development, University of Zurich, Andreasstrasse 15, 8050 Zurich, Switzerland; 2 Department of Sociology, University of Zurich, Andreasstrasse 15, 8050 Zurich, Switzerland; 3 Social Research Institute, Institute of Education, University College London, London, UK

**Keywords:** Individual agency, Social origin, Educational transitions, Tracking, Longitudinal

## Abstract

Little is known about the role of agency in transitions in tracked education systems or whether it varies by socioeconomic background. This study addressed this gap by estimating structural equation models based on longitudinal data that are representative of the German- and French-speaking parts of Switzerland (*N* = 1273 individuals, surveyed from age 6 to 18, mean age at wave 1: *M*_age_ = 6.54, SD_age_ = 0.50, female = 49%). The findings reveal that agency (captured by study effort and occupational aspirations) and socioeconomic background (measured by parental education and family income) significantly predicted students’ transitions to academically demanding tracks in lower- and upper-secondary education. In the transition to upper-secondary education, students with fewer socioeconomic resources benefitted less than their more advantaged peers from ambitious aspirations, but they benefitted more from exerting effort. These findings suggest that both an optimistic forward-looking orientation and the exertion of effort are required to make it to an academic track. Effort may serve as a “substitutive” resource for less socioeconomically advantaged students, whereas ambitious aspirations may enhance the positive effect of family socioeconomic resources on academic educational trajectories. Overall, the evidence from this study calls for greater attention to investigating not only how agency shapes adolescents’ educational trajectories and opportunities but also how its role differs across social groups.

## Introduction

Early transitions in tracked education systems are crucial in shaping individual educational trajectories. To transition to academically demanding tracks, students need to exert agency (Buchmann & Steinhoff, 2017); they must exert effort (Burger, 2021) and orient themselves toward a given future goal to motivate their action (Holtmann et al., 2021). Their families typically require socioeconomic resources to support their children’s academically oriented educational trajectories (Blossfeld et al., 2016). Socioeconomic resources may also influence the extent to which individual agency can facilitate the transition to an academically demanding track, either strengthening or weakening its effect (Schoon & Cook, 2021). However, there has been insufficient empirical research into the role that individual agency can play in shaping educational transitions in tracked education systems. Moreover, there has been little research on how agency shapes multiple sequential educational transitions in such systems (e.g., Steinhoff & Buchmann, 2017), and there is a paucity of studies on the interplay between agentic and socioeconomic resources at early transitions (e.g., Gil-Hernández, 2021). Against this background, this study sought to untangle the interplay between agency and socioeconomic resources in the Swiss education system, which partially channels educational trajectories along tracks with distinct levels of academic requirements from lower-secondary school, that is, age 12, onward.

### Individual Agency at Transitions

Education systems that use tracking allocate students to educational programs with different academic demands based on a mix of teachers’ recommendations, ability testing (e.g., previous grades, admission tests), and parental choices (Brunello & Checchi, 2007). This type of tracked system sorts students at times of transitions—the “junctures” of the system—when students move from one educational level to another (Burger, 2021). Typically, these transitions occur in early adolescence, a life period that is marked by many potentially challenging developmental processes, the exploration of new social roles, and important biological changes, with potentially wide-ranging consequences for later life-course outcomes (Buchmann & Kriesi, 2011). Once students have accessed a certain educational path, most tend to follow a typical sequence of transitions (Hillmert & Jacob, 2010). However, students do not transition to academically demanding tracks automatically. Individual agency is required to make such a transition.

Agency broadly refers to the individual’s capacity to act and influence their own life course—self-initiated action (e.g., Gecas, 2003). Agency has been widely conceptualized as a construct that entails different temporal orientations towards the present and the future (Hitlin & Elder, 2007). Whereas the most common conceptualizations of agency have focused on the capacity to act in the present, more recently scholars have stressed the importance of also accounting for temporally-extended dimensions of agency (Hitlin & Johnson, 2015). Through the exercise of forethought, individuals orient themselves toward a future goal and motivate their actions. The anticipation of future events provides direction and coherence toward accomplishing the desired goals (Frye, 2012). Though situational and forward-looking dimensions of agency may be linked to each other (Domina et al., 2011), they do not strictly presuppose each other; on the contrary, they may be partly independent of each other and, as such, should be analytically separated (Hitlin & Elder, 2007). This study focuses on a situational, present-oriented dimension of agency—study effort—and a more forward-looking dimension of agency—occupational aspirations. Successfully transitioning to academically demanding tracks may require both (Schoon & Ng-Knight, 2017).

Occupational aspirations are defined as goals or desires regarding individuals’ future occupations (Basler & Kriesi, 2019). They express an orientation towards a future desired occupational position, capturing the long-term time horizon of individual agency. Occupational aspirations have been widely found to positively predict educational outcomes in multiple contexts (e.g., Beal & Crockett, 2010). However, future aspirations alone may be insufficient if students lack the capacity to exert effort in pursuing such goals (Schoon & Heckhausen, 2019). Projecting towards a future goal also requires the ability to regulate and control its execution by taking action in the present, for instance, in terms of study effort. Study effort refers to the intensity of the commitment to studying and the amount of energy invested toward achieving an academic goal (Rieger et al., 2022). Not surprisingly, scholars have been able to document the positive influence of effort on educational outcomes (Palacios-Abad, 2021). Effort has been identified among the dimensions of agency holding a primary role in the motivational system, being especially stable over the life course. It is beneficial for immediate and short-term tasks and in many everyday situations (Heckhausen et al., 2010). Hence, study effort represents a key agentic resource in educational transitions.

### Socioeconomic Resources at Transitions

Socioeconomic resources available in the family are recognized as an influence on students’ probabilities to transition to academically demanding tracks (Neugebauer et al., 2013). Referring to the classical distinction between economic, cultural, and social resources—coined *capital*—this study focuses on the two key dimensions of economic and cultural resources (Bourdieu, 1984). Cultural capital refers to the accumulation of education in the home environment, the physical presence of cultural objects, and specific preferences. More cultural resources accrued in the family may lead to a more stimulating home learning environment, better guidance in educational decisions, and a better understanding of the functioning, norms, and codes of behavior in the education system (Ditton et al., 2019). The present study captures familial cultural resources via parental education, in line with the existing literature (Burger & Walk, 2016). Economic capital entails the material and financial assets available in the family (Jæger, 2007). Economic resources may enable parents to better support their children in their studies through tutoring, purchasing educational material, or providing adequate space to study, while children’s perceptions of economic pressure and financial stress may have a negative impact on their academic outcomes (Mistry & Elenbaas, 2021). Furthermore, the perception of greater economic security may encourage educational choices that require staying longer in education and delaying entrance into the labor market, such as choices towards more academically oriented trajectories (Blanden & Gregg, 2004). The current study investigates parental economic resources through family income, as has commonly been done in prior research (Jæger & Holm, 2007).

Parental education and family income (hereafter also referred to as *socioeconomic resources* for the sake of brevity) have well-documented benefits for educational attainment (Pensiero & Schoon, 2019) via processes of socialization and educational choices (Breen & Jonsson, 2005). They have proven to be especially beneficial for students in tracked systems in meeting the requirements of academically demanding tracks (Leemann et al., 2022). Importantly, familial socioeconomic resources appear to be strongly associated with educational attainments at early educational transitions (Hillmert & Jacob, 2010), which may be especially dominated by the direct influence of parents due to the young age of students (Heckhausen, 2021).

### The Interplay between Agency and Socioeconomic Resources at Transitions: Resource “Substitution” and “Multiplication”

Family socioeconomic resources may also moderate the influence of individual agency on students’ probabilities of transitioning to a given educational track (Heckhausen & Shane, 2015). The extent to which students can use agency in pursuing their educational trajectories is “bounded” by external circumstances, such as socioeconomic background (Shanahan, 2000). However, a better understanding is needed of how agency interacts with socioeconomic background in shaping educational transitions.

Scholars point to two alternative mechanisms that may characterize the interplay between individual agency and socioeconomic resources: *resource multiplication* and *resource substitution* (Ross & Mirowsky, 2006). The *resource multiplication* process occurs when available resources multiply each other’s impact. Children from socioeconomically advantaged families may also reap larger benefits from their agentic resources, amplifying their overall advantage over less privileged children when engaging in educational transitions. The *resource substitution* process takes place when the presence of one resource substitutes for the absence of another, making the latter less disruptive. Children from less advantageous backgrounds, counting on fewer socioeconomic resources, may rely more heavily on agentic resources in educational transitions. Their agentic resources could function as a viable substitute for family socioeconomic resources.

A recent line of research has started to test these competing hypotheses regarding “resource multiplication” and “substitution”. Evidence has been found for “resource multiplication” effects between agency and socioeconomic resources (Brumley et al., 2019; Kwon & Erola, 2022) as well as “resource substitution” processes (Johnson & Hitlin, 2017; Schoon, 2014). However, the interplay between agency and socioeconomic resources may vary across educational transitions (Liu, 2019). This could be the case if the influence of agency on educational outcomes increases from early childhood to adolescence. The diverging findings may also be explained by different dimensions of agency interacting differently with socioeconomic resources (Holtmann et al., 2021; Lee & Mortimer, 2021).

## Current Study

Little evidence exists on the roles of individual agency and socioeconomic resources, as well as their interplay in the early stages of individuals’ educational trajectories. Against this background, this study sought to explore these mechanisms by focusing on the Swiss education system, an ideal case to investigate early educational transitions because in this system, students are assigned to different tracks with distinct levels of academic requirements from lower-secondary school onward. The study focused on the transitions to academically demanding tracks (from now on, for the sake of brevity, also referred to as *academic tracks*) in lower- and upper-secondary education. The two transitions of interest were studied simultaneously to account for the strong link between transitions characterizing highly tracked systems. Prior research has identified study effort and occupational aspirations as two crucial dimensions of individual agency, tapping into different time horizons which are both needed at times of transitions. Building on this research, the first aim of the current study was to capture their separate roles in early transitions. More specifically, the first hypothesis was that agency in terms of study effort and occupational aspirations is positively associated with the probability of transitioning to an academic track in lower- and upper-secondary education (Hypothesis 1). Moreover, relying on the abundant evidence regarding the role of familial cultural and financial resources in educational transitions, the second hypothesis was that family socioeconomic resources (parental education and family income) are positively associated with the probability of transitioning to an academic track in lower- and upper-secondary education (Hypothesis 2). Finally, drawing on theory about “resource multiplication” and “resource substitution” effects, this study explored the interplay between agency and two familial socioeconomic resources—parental education and family income. Specifically, the third hypothesis posited that socioeconomic resources moderate the link between children’s agency and their probability of transitioning to academic tracks (“resource multiplication” or “resource substitution”) (Hypothesis 3).

## Methods

### Data and Sample

Data were used from the multi-informant Swiss Longitudinal Survey of Children and Youth (COCON), which followed a cohort of children born in 2000. The survey is representative of the French- and German-speaking parts of Switzerland (which at the time of data collection made up 92% of the Swiss population). The sample was selected in a two-stage procedure from 131 communities: first, the communities were selected; second, children were randomly drawn from the communities’ official resident registers (Buchmann et al., 2021). The initial response rate among the selected households was 78%. The analyses incorporated information collected from children at the age of 6, 9, 12, 13, 15, 16, and 18. This study used information reported by children and primary caregivers (mainly mothers). Data were collected using computer-assisted personal interviews (CAPI) and computer-assisted telephone interviews (CATI). Additionally, oral interviews with primary caregivers were supplemented by printed questionnaires administered from 2006 until 2015. All measures used in this study were collected from children, except for family income, parental education and mother tongue, which were reported by the primary caregiver. All measures are based on closed-ended questions and reflect self-reports. The sample included 1273 children in the first wave, among which 51% were male; 17% had a mother tongue different from the official Swiss languages (see Table 1).

**Table 1:** Descriptive statistics

Measures	Measured in	Mean	SD	Min.	Max.	*N*
Family background						
Parental tertiary education	2006	0.50	–	0	1	1272
Family income (log)	2006–2015	11.33	0.48	9.20	12.01	1215
Respondent at age 6						
Male	2006	0.51	–	0	1	1273
Foreign language	2006	0.17	–	0	1	1218
Cognitive ability	2006	2.35	1.60	0	6	1273
Respondent at age 12						
Study effort	2012					
Apply myself to study/work		4.20	1.03	1	6	1034
Try hard at school/work		4.64	0.93	1	6	1033
Do what is necessary for school/work^a^		3.54	1.30	1	6	1034
Occupational aspirations^b^	2012	5.39	1.95	2.3	8.8	802
Early transition	2012/2013	0.27	–	0	1	852
Respondent at age 14/15						
Academic track attended in lower-secondary education	2014/2015	0.55	–	0	1	925
Long-term baccalaureate	2015	0.09	–	0	1	935
Study effort	2015					
Apply myself to study/work		3.72	1.17	1	6	930
Try hard at school/work		4.44	0.97	1	6	930
Do what is necessary for school/work^a^		3.37	1.28	1	6	929
Occupational aspirations^b^	2015	5.33	1.86	2.2	8.8	812
Respondent at age 16–18						
Academic track attended in upper-secondary education	2016–2018	0.35	–	0	1	808
Delayed transition	2016–2018	0.22	–	0	1	808

^a^The item was reverse-coded from the original formulation “For school/work I only do what is necessary” (1 = “I absolutely do not agree”; 6 = “I completely agree”)

^b^The variable was rescaled from the original range 16–90 to 1.6–9 to harmonize it with the other variables

### Measures

#### Track Attended in Lower-Secondary Education

The study distinguished academically demanding tracks (coded as 1) from less academically demanding tracks (coded as 0).

#### Track Attended in Upper-Secondary Education

The study distinguished the academic track (coded as 1) that prepares students for tertiary education in universities from vocational or general education tracks (coded as 0) that do not prepare students for tertiary education in universities.

#### Study Effort

Study effort was measured using a latent construct estimated with three items that captured self-reported study effort at the age of 12 and 15, before students transitioned to lower- and upper-secondary education. The items were: “When I study/work I apply myself as much as possible”, “I try very hard at school/work”, and “For school/work I only do what is necessary” (reverse-coded) (adapted from Moser, 1997), measured on a scale from 1 (“does not apply”) to 6 (“fully applies”) (McDonald’s omega = 0.61 for the age 12 scale and = 0.77 for the age 15 scale).

#### Occupational Aspirations

Participants’ occupational aspirations were measured at the age of 12 and 15 using the item “Which job/profession do you aspire to have in the future?” and converting the responses to the respective score on the international socioeconomic index of occupational status scale (ISEI scores) (Ganzeboom et al., 1992). The scale ranges from 16 (lowest-status occupational aspirations) to 90 (highest-status occupational aspirations). To harmonize the scale with the other variables, it was rescaled to range from 1.6–9.

#### Family Income

Family income is based on a measure that captures the household’s annual net income at the age of 6, 9, 12, and 15, distinguishing between 7 categories ranging from less than 20,000 (1) to more than 150,000 CHF (7). The mid-points were calculated for each category and the values were adjusted for inflation by converting all income levels to 2015 equivalents (as the measure was collected from 2006 to 2015) (Burger et al., 2020). The adjustments were based on the Consumer Price Index (Federal Statistical Office, 2021). The obtained values were averaged across waves and transformed using the natural logarithm to account for a decrease in the marginal utility of income for higher-income families.

#### Parental Education

The highest parental educational attainment was assessed in the first wave when the children were 6 years old. The measure captures the highest level of education attained among both parents, distinguishing between parents who had completed a degree in tertiary education (obtained from a University of Applied Sciences or University/Federal Institute of Technology) (coded as 1) from those who held an upper-secondary education degree maximally (coded as 0).

#### Controls

To better capture the effects of interest, dichotomous variables were added to control for the timing of transitions. At the time of data collection, in most cantons, primary education encompassed 6 years of schooling; in some cantons, primary education covered 5 years, so children typically transitioned to lower-secondary education one year before (between the age of 11 and 12) their peers in the other cantons (between the age of 12 and 13). This variation was captured by adding a control for an *early transition*, distinguishing those children who at the age of 12 were already attending lower-secondary education (coded as 1) from all their peers in the other cantons (coded as 0). As some cantons offer a long-term academic baccalaureate program (“Langzeitgymnasium”) beginning at the lower-secondary education level, a control was also added for *long-term baccalaureate* (coded as 1). Finally, a control was added for those students who experienced a *delayed transition* to upper-secondary education (coded as 1) to capture those adolescents who completed this transition after the “normative” age of 16. *Cognitive ability* was assessed when children were 6 years old using the basic non-verbal intelligence test (CFT 1) (Cattell et al., 1977). *Gender* was assessed when children were 6 years old, distinguishing between male (coded as 1) and female (coded as 0). The *mother tongue* of children was collected when children were 6 years old and was used in this study as a proxy for migrant background. The study distinguished children with a foreign language (coded as 1) from those who spoke one of the official Swiss languages (coded as 0).

### Analytical Strategy

**Fig. 1:**
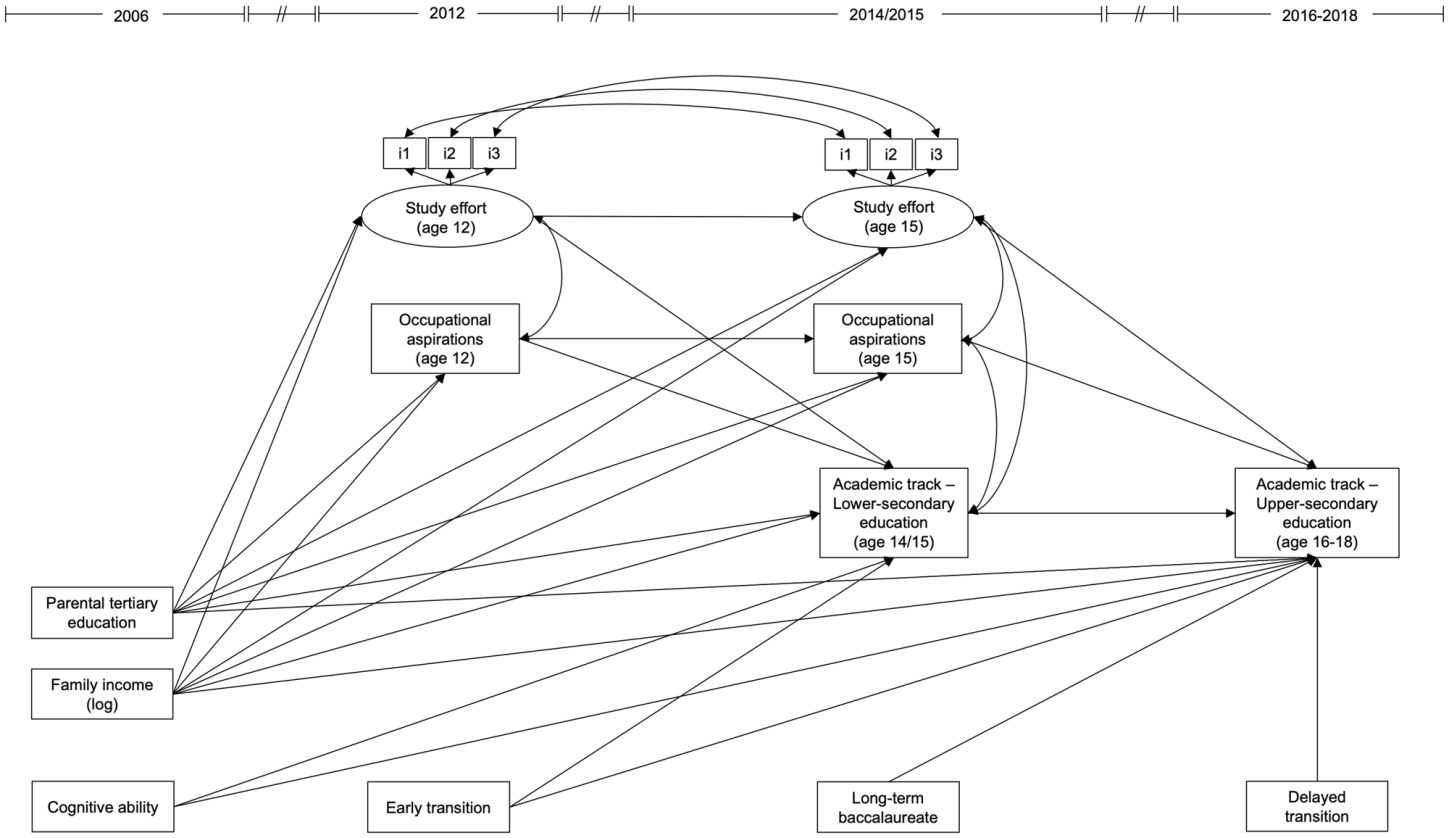
Full main effects model specification. For brevity, controls for male and foreign language are not shown but are applied in the same manner as parental tertiary education and family income. Similarly, residual correlations among exogenous variables are not displayed

The analysis followed a three-stage procedure to test the hypotheses. First, to assess the association between agentic and socioeconomic resources and the probability of transitioning to academic tracks at the two educational transitions (Hypothesis 1 and Hypothesis 2), the study used a longitudinal structural equation model (SEM) with a linear specification (Little, 2013). SEM provides several advantages, making this strategy particularly suited for the purpose of this study. It enables a reduction of measurement error via the estimation of latent factors, and enables researchers to assess multiple hypotheses simultaneously and also determine residual correlations among study variables (Ullman & Bentler, 2012). Estimating residual correlations, in particular, allows for more precise and unbiased estimates of the parameters of interest (Little, 2013) while simultaneously shedding light on additional patterns of relationships among the study variables next to the main parameters of interest. Study effort was measured as a latent construct. To ensure that the same construct was measured across time, the measurement invariance of study effort was tested (Cole & Maxwell, 2003). Results from a set of confirmatory factor analyses with increasing constraints on factor loadings and intercepts indicated configural, metric, and scalar invariance (see Online Resource S2 in the supplementary materials for further details about these analyses). The residual variances of correspondent items of study effort measured at the age of 12 and 15 were allowed to correlate, based on the assumption that the reliable residual variance of correspondent indicators is likely to correlate across time (Little, 2013). Within-time residual correlations were specified among study variables (Cole & Maxwell, 2003) (see Fig. 1). To ensure parsimony, the model only included significant relationships among study variables (as recommended by Little, 2013), leaving across-time residual correlations among parallel items’ residuals and within-time residual correlations in the model, even if non-significant (as recommended by Cole & Maxwell, 2003; Little, 2013). Autoregressive paths were specified to assess whether occupational aspirations and study effort are stable over time (Cole & Maxwell, 2003) (see Fig. 1). Cluster-robust standard errors were estimated to account for the nesting of students in different educational tracks (Cameron & Miller, 2015). Finally, model fit was assessed using the most commonly used goodness-of-fit measures in SEM, Comparative Fit Index (CFI), Tucker-Lewis Index (TLI), and Root Mean Square Error of Approximation (RMSEA), as each of these indices has a different sensitivity to model misspecifications (Hu & Bentler, 1998). Model fit was considered acceptable when CFI > 0.90, TLI > 0.90, and RMSEA < 0.08 (Little, 2013).

Second, the moderation hypothesis (Hypothesis 3) was tested. Four interaction terms were entered between family income and the two agency constructs at the two transitions into the model (Model 2). Next, the procedure was replicated for the moderation by parental education, entering the correspondent four interaction terms with parental education into a separate model (Model 3). The interaction terms were built by employing the two-stage ordinary least square procedure of residual centering (Little et al., 2007) to ensure full independence between the interaction terms and the first-order variables. This allowed for interpreting the main effects and obtaining unbiased and more stable estimates of the interaction terms and first-order variables (Little et al., 2006). Additionally, whereas the interaction terms with occupational aspirations build on manifest variables, latent interaction terms were used with study effort, providing greater statistical power (Steinmetz et al., 2011) (see Online Resource S3 for details about the procedure). To aid the interpretation of the moderation analysis, the results were also visualized as predicted probabilities estimated from linear probability models (LPM) (the details of the procedure are reported in the Online Resource S6). This allowed for identifying how the transition probabilities vary as the values of the key variables of interest change.

The sample of 1273 participants showed 38% attrition in the last wave analyzed (age of 18) compared to the initial wave (age of 6). Logistic regressions revealed that dropping out of the study was less likely among children from higher-educated families (odds ratio [OR] = 0.74, *p* < 0.05) and higher-income families (OR = 0.57, *p* < 0.001), and more likely among children speaking a foreign language (OR = 2.02, *p* < 0.001). The percentage of missing values on the variables included in the model ranged from 0 to 36.5% (nonresponse across items and waves). To correct for potential bias related to the presence of missing data, full information maximum-likelihood (FIML) estimation was used in the structural equation models. FIML uses all the available data in the model, it determines the estimates that reduce the standardized distance to the observed data (Enders, 2010). When missingness is related to observed variables incorporated in the analysis model, as in the present case, FIML generates more unbiased estimates of parameters than more traditional techniques such as listwise or pairwise deletion (Baraldi & Enders, 2010). All analyses were conducted in STATA 17 (StataCorp, 2021). Figures were also produced using STATA 17.

## Results

### Descriptive Results

**Fig. 2:**
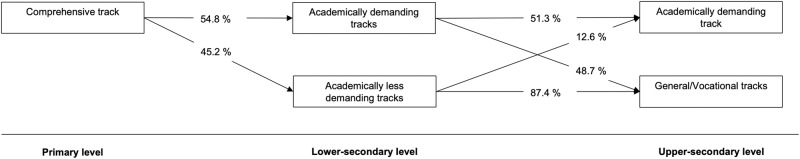
Percentages of students transitioning to distinct educational tracks

The descriptive statistics of all study variables are presented in Table 1. Pairwise correlations among study variables are shown in Table S1 in the supplementary materials. Figure 2 summarizes the percentages of students who transitioned from primary education to either academically demanding tracks or less academically demanding tracks in lower-secondary education, and from there to either the academic track or general or vocational education at the upper-secondary level. 54.8% of students transitioned to academic tracks in lower-secondary education and around half of them eventually continued to the academic track in upper-secondary education. Around 13% of students in less academically demanding tracks in lower-secondary education managed to enter the academic track in upper-secondary education, while 87.4% transitioned into general or vocational education.

### Results from Structural Equation Models

**Fig. 3:**
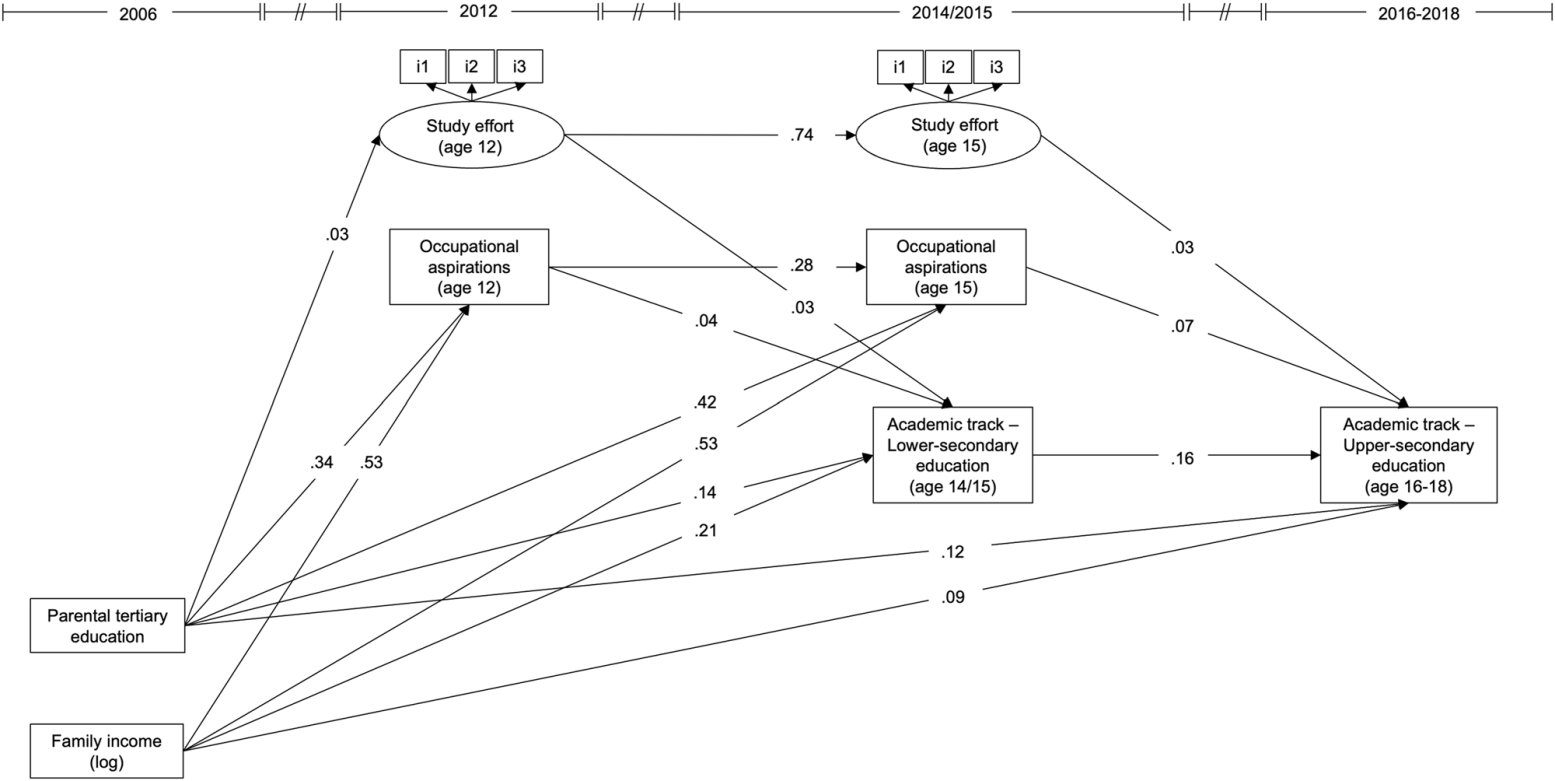
Structural equation model without interactions (Model 1). Coefficients generated from a linear probability SEM. Coefficients should be interpreted net the other effects estimated in the model. Unstandardized coefficients are reported, enabling the interpretation of the results in their original metric. The remaining significant paths for the covariates—male, foreign language, cognitive ability, and controls for timing of the transitions—are not shown here for the sake of readability (see Table 2)

Turning to the results of the structural equation models and assessing the hypotheses, Table 2 shows the unstandardized and standardized coefficients and model fit statistics from the structural equation models. Table 3 shows the factor loadings and residual correlations and Table S3 in the supplementary materials shows the correspondent models that retain non-significant paths. Figure 3 illustrates the main results from Model 1.

**Table 2:** Path coefficients and model fit statistics from the structural equation models

		Model 1		Model 2		Model 3	
Outcome	Predictor	Coeff. (Std. coeff.)	SE	Coeff. (Std. coeff.)	SE	Coeff. (Std. coeff.)	SE
Occupational aspirations (age 12)							
	Parental tertiary education	0.339*** (0.088)	0.028	0.337*** (0.087)	0.027	0.337*** (0.087)	0.027
	Family income (log)	0.529*** (0.122)	0.025	0.529*** (0.122)	0.025	0.530*** (0.122)	0.026
	Foreign language	1.101*** (0.188)	0.001	1.106*** (0.189)	0.002	1.105*** (0.189)	0.001
Study effort (age 12)							
	Male	−0.616*** (−0.294)	0.105	−0.616*** (−0.294)	0.105	−0.616*** (−0.294)	0.105
	Parental tertiary education	0.033*** (0.016)	0.006	0.034*** (0.016)	0.006	0.034*** (0.016)	0.006
Academic track – Lower-secondary							
	Occupational aspirations (age 12)	0.042*** (0.163)	0.001	0.042*** (0.164)	0.001	0.042*** (0.163)	0.001
	Study effort (age 12)	0.034** (0.071)	0.011	0.034** (0.071)	0.011	0.034** (0.071)	0.011
	Parental tertiary education	0.135*** (0.136)	0.035	0.136*** (0.136)	0.035	0.135*** (0.136)	0.035
	Family income (log)	0.211*** (0.190)	0.023	0.211*** (0.189)	0.023	0.211*** (0.190)	0.023
	Foreign language	−0.079*** (−0.053)	0.015	−0.080*** (−0.053)	0.015	−0.079*** (−0.053)	0.015
	Cognitive ability	0.028*** (0.088)	0.004	0.027*** (0.088)	0.004	0.028*** (0.088)	0.004
	Early transition	0.085*** (0.075)	0.017	0.085*** (0.075)	0.018	0.084*** (0.074)	0.019
Occupational aspirations (age 15)							
	Occupational aspirations (age 12)	0.282*** (0.290)	0.027	0.282*** (0.290)	0.027	0.282*** (0.290)	0.027
	Parental tertiary education	0.423* (0.112)	0.191	0.423* (0.112)	0.194	0.422* (0.112)	0.189
	Family income (log)	0.530* (0.126)	0.234	0.530* (0.126)	0.241	0.530* (0.126)	0.236
	Foreign language	0.312*** (0.055)	0.009	0.310*** (0.054)	0.000	0.310*** (0.054)	0.000
Study effort (age 15)							
	Study effort (age 12)	0.741*** (0.552)	0.035	0.741*** (0.552)	0.036	0.742*** (0.552)	0.036
	Male	−0.238*** (−0.085)	0.003	−0.238*** (−0.085)	0.003	−0.238*** (−0.085)	0.003
	Foreign language	0.247*** (0.058)	0.010	0.249*** (0.058)	0.014	0.248*** (0.058)	0.011
Academic track – Upper-secondary							
	Academic track – Lower-secondary	0.164*** (0.176)	0.043	0.165*** (0.177)	0.031	0.168*** (0.180)	0.037
	Occupational aspirations (age 15)	0.074** (0.299)	0.026	0.073*** (0.298)	0.021	0.073** (0.297)	0.023
	Study effort (age 15)	0.030** (0.092)	0.011	0.030*** (0.092)	0.008	0.031*** (0.095)	0.007
	Parental tertiary education	0.115*** (0.124)	0.027	0.117*** (0.126)	0.032	0.112*** (0.120)	0.015
	Family income (log)	0.094** (0.091)	0.035	0.095*** (0.091)	0.003	0.097** (0.093)	0.031
	Foreign language	0.070** (0.050)	0.026	0.069** (0.049)	0.025	0.065*** (0.046)	0.018
	Cognitive ability	0.032** (0.111)	0.010	0.032** (0.110)	0.010	0.032** (0.110)	0.010
	Long-term baccalaureate	0.404*** (0.257)	0.051	0.397*** (0.252)	0.041	0.397*** (0.251)	0.039
	Occupational aspirations (age 15) × Family income			0.041*** (0.074)	0.006		
	Study effort (age 15) × Family income			−0.013*** (−0.029)	0.003		
	Occupational aspirations (age 15) × Parental education					0.037** (0.073)	0.014
	Study effort (age 15) × Parental education					−0.014** (−0.030)	0.005
Model fit statistics							
*N*		925		925		925	
χ2 (*df*)		338.82 (98)		604.66 (242)		574.58 (247)	
CFI		0.900		0.912		0.913	
TLI		0.872		0.898		0.901	
RMSEA (90% CI)		0.044 (0.039;0.049)		0.034 (0.031;0.038)		0.032 (0.029;0.036)	

*Std.* Standardized. *SE* Cluster-robust standard errors. Goodness-of-fit measures are obtained by estimating the models using the Observed Information Matrix estimator. Unstandardized coefficients are useful for interpreting binary variables whereas standardized coefficients are useful for interpreting continuous variables. Items for study effort were standardized before being included in the model (as in Schoon & Ng-Knight, 2017). Because of the clustering of standard errors by school tracks, the final sample used in the analysis counts 925 observations corresponding to the total number of observations in the different tracks. *df* degrees of freedom. *CFI* comparative fit index. *TLI* Tucker-Lewis index. *RMSEA* Root mean square error of approximation. *CI* confidence interval

*p < 0.05; **p < 0.01; ***p < 0.001

**Table 3:** Standardized factor loadings and residual correlations among study variables from the structural equation models

		Model 1	Model 2	Model 3
Latent factors	Indicators	Standardized loading	Standardized loading	Standardized loading
Study effort (age 12)				
	Apply myself to study/work	0.568***	0.567***	0.568***
	Try hard at school/work	0.469***	0.468***	0.468***
	Do what is necessary for school/work	0.545***	0.546***	0.546***
Study effort (age 15)				
	Apply myself to study/work	0.753***	0.752***	0.752***
	Try hard at school/work	0.632***	0.632***	0.632***
	Do what is necessary for school/work	0.741***	0.742***	0.742***

Residual correlation between	and	Standardized coefficients	Standardized coefficients	Standardized coefficients
Male	Cognitive ability	−0.063***	−0.065**	−0.065***
Male	Delayed transition	0.095***	0.095***	0.101***
Male	Long-term baccalaureate	–	–	−0.048*
Parental tertiary education	Family income (log)	0.449***	0.446***	0.445***
Parental tertiary education	Foreign language	−0.053*	–	–
Parental tertiary education	Cognitive ability	0.173***	0.172***	0.176***
Parental tertiary education	Early transition	0.068***	0.061***	0.064***
Parental tertiary education	Delayed transition	−0.216*	−0.219*	−0.219*
Parental tertiary education	Long-term baccalaureate	0.182**	0.182***	0.180***
Family income (log)	Foreign language	−0.119***	−0.098***	−0.097***
Family income (log)	Cognitive ability	0.159***	0.161***	0.161***
Family income (log)	Early transition	0.151**	0.149**	0.155**
Family income (log)	Delayed transition	−0.188***	−0.191***	−0.189***
Family income (log)	Long-term baccalaureate	0.155***	0.152***	0.153***
Foreign language	Cognitive ability	–	−0.041*	–
Foreign language	Early transition	−0.117***	−0.120***	−0.115***
Cognitive ability	Early transition	0.099***	0.103***	0.099***
Cognitive ability	Delayed transition	−0.192***	−0.191***	−0.193***
Cognitive ability	Long-term baccalaureate	0.078***	0.078***	0.080***
Occupational aspirations (age 12)	Study effort (age 12)	0.137	0.137	0.137
Occupational aspirations (age 15)	Study effort (age 15)	0.091	0.091	0.092
Academic track - Lower-secondary	Occupational aspirations (age 15)	0.198***	0.198***	0.198***
Academic track - Lower-secondary	Study effort (age 15)	−0.036*	−0.035*	−0.036*
Delayed transition	Long-term baccalaureate	−0.169**	−0.167**	−0.171**

Significance levels refer to unstandardized estimates. Reported factor loadings are estimated from measurement models that use the fixed factor scaling method (as recommended by Little, 2013) where all factor variances are constrained to 1 and factor means to 0. (-) identifies the cells where the corresponding residual correlation was pruned in the model because non-significant. Correlations among items’ residual variances, factor loadings of interaction terms, residual correlations among interaction terms, and between interaction terms and other variables are not displayed

*p < 0.05; **p < 0.01; ***p < 0.001

In line with the first hypothesis, Model 1 suggests that study effort and occupational aspirations were significantly and positively related to individuals’ probabilities of attending an academically demanding track in lower-secondary education (*β* = 0.034, *p* < 0.01; and *β* = 0.042, *p* < 0.001, respectively) and the academic track in upper-secondary education (*β* = 0.030, *p* < 0.01; and *β* = 0.074, *p* < 0.01, respectively).

The results also supported Hypothesis 2, which posited that parental education and family income would be positively associated with the probability of transitioning to academic tracks in lower- and upper-secondary education. Compared to students with lower-educated parents (i.e., maximum of upper-secondary education), students with higher-educated parents (i.e., tertiary education) were 13.5 percentage points more likely to transition into an academic track at the lower-secondary education level (*p* < 0.001) and 11.5 percentage points more likely to transition to the academic track at the upper-secondary education level (*p* < 0.001). Family income (logged) was positively associated with the probabilities of transitioning to an academic track at the lower-secondary education level (*β* = 0.211, *p* < 0.001) and to the academic track at the upper-secondary education transition (*β* = 0.094, *p* < 0.01).

Finally, the moderation hypothesis (Hypothesis 3) can be scrutinized. The interaction terms between individual agency and family income (Model 2) and between individual agency and parental education (Model 3) were examined (see Fig. S2 in the supplementary materials for a summary of the main results). Models 2 and 3 showed no evidence of significant interactions between individual agency and family income or between agency and parental education in the transition to lower-secondary education (see Table S3 in the supplementary materials for models retaining non-significant interactions). Hypothesis 3 was not supported for the transition to lower-secondary education. By contrast, the models indicated statistically significant interactions between children’s agency and socioeconomic resources in the transition to the academic track in upper-secondary education, in line with Hypothesis 3. Models 2 and 3 revealed that among children in families with greater socioeconomic resources, the link between children’s occupational aspirations and their probability of transitioning to the academic track at the upper-secondary level was significantly stronger than for children with fewer socioeconomic resources (*β* = 0.041, *p* < 0.001; and *β* = 0.037, *p* < 0.01, respectively). These results point to a “resource multiplication” effect. Second, the models indicated that among children with greater socioeconomic resources, the link between study effort and the probability of transitioning to the academic track in upper-secondary education was significantly weaker than among those with fewer socioeconomic resources (*β* = −0.013, *p* < 0.001; and *β* = −0.014, *p* < 0.01, respectively). These results point to a “resource substitution” effect.

**Fig. 4:**
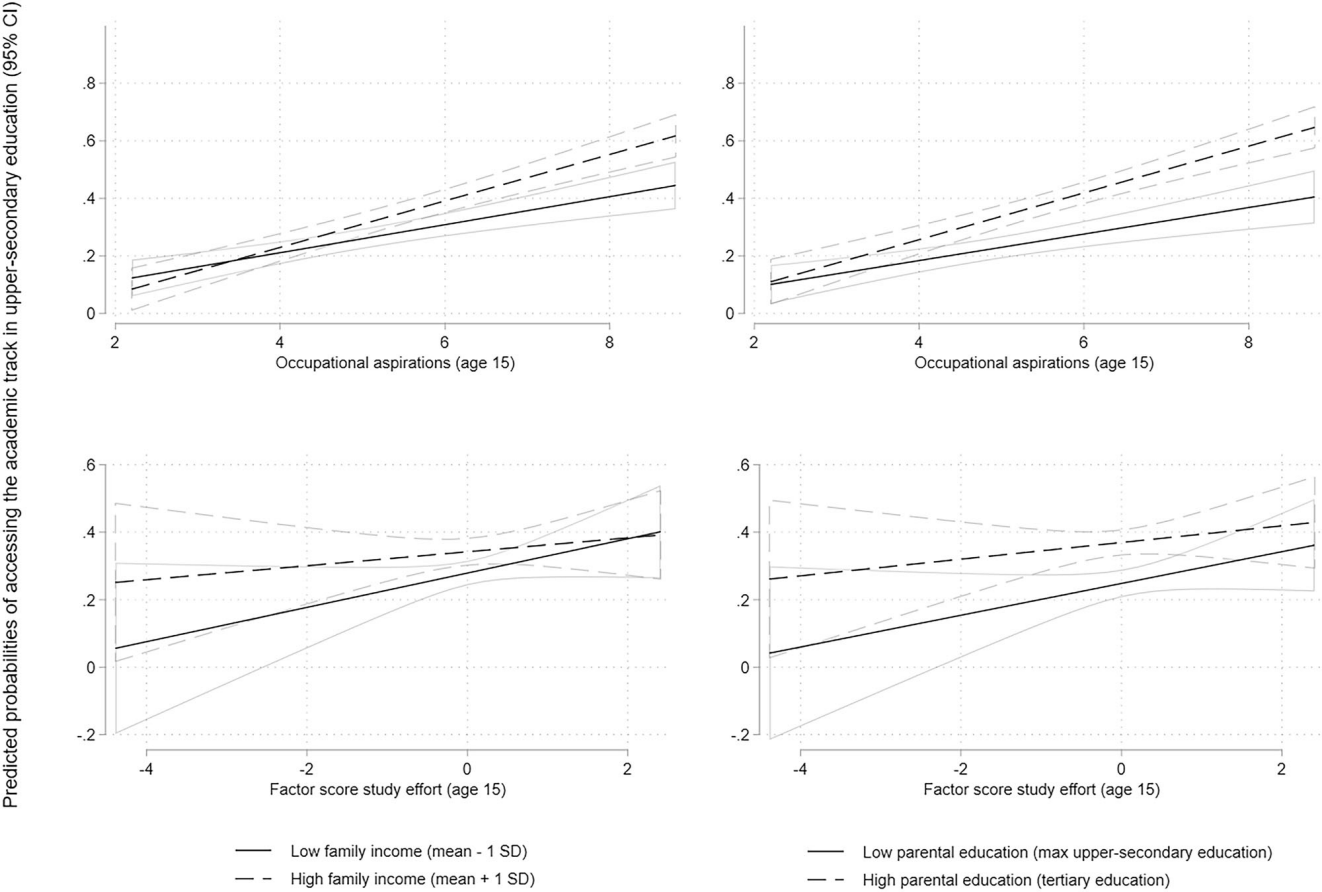
Predicted probabilities of transitioning to the academic track in upper-secondary education as a function of occupational aspirations (top row) and study effort (bottom row), at fixed values of family income (left column) and parental education (right column). Figures are based on individual LPMs and not on the entire SEM; this might explain the minor differences compared to Models 2 and 3. SD Standard deviation

To aid the interpretation of the four significant interactions, the predicted probabilities of transitioning to the academic track in upper-secondary education were estimated and plotted in Fig. 4 as a function of the two measures of agency at fixed values of family income (1 SD above and below the mean) and parental education (maximum upper-secondary education versus tertiary education), while holding all other covariates constant. Because the sample included a nonnegligible proportion of children (roughly 13%) who transitioned from less academically demanding tracks in lower-secondary education to the academic track in upper-secondary education, the average effects across students in the two tracks in lower-secondary education were calculated. Figure 4 confirms the findings from Models 2 and 3. The charts show that among children from families with greater socioeconomic resources, the link between children’s occupational aspirations and their probability of transitioning to the academic track at the upper-secondary level was significantly stronger than among those with fewer socioeconomic resources (only minor differences are visible between the predicted probabilities by family income and parental education). By contrast, the figure indicates that among children with greater socioeconomic resources, the link between study effort and the probability of transitioning to the academic track in upper-secondary education was weaker than among those with fewer socioeconomic resources (only negligible differences are detectable, also in this case, between the predicted probabilities by family income and parental education).

### Sensitivity Analyses

Even though the factor loadings for study effort at the age of 12 were acceptable (Briggs & MacCallum, 2003), they were relatively weak, potentially signaling issues in the measurement model. Such issues would pose a threat to construct validity and introduce bias when estimating the structural relationships of interest (Rhemtulla et al., 2020). To test the robustness of the structural parameters against these potential threats, Model 1 was replicated using a composite score (obtained by averaging items’ scores) to measure study effort instead of a latent variable. A composite score was used because the parameters generated by models with composite scores tend to be less sensitive to the strength of the correlations among the indicators, compared to parameters from models that use latent constructs. Such models are also likely to be more robust to the variability of the associations between indicators and other variables in the model (Rhemtulla et al., 2020). Results from this analysis did not differ substantially from those obtained from Model 1. With regard to the interaction effects in the transition to upper-secondary education, additional analyses were performed to test whether the results were sensitive to the educational track that students attended in lower-secondary school. The results from the main analysis were replicated, estimating predicted probabilities for students who attended distinct tracks in lower-secondary education. These findings are reported in Fig. S4 in the supplementary materials. Despite minor differences, the results from this additional analysis confirmed the findings.

## Discussion

The exertion of individual agency at early stages of educational trajectories in tracked education systems may be a key resource for youth to influence their later educational and occupational opportunities. However, little is known about how individual agency shapes educational transitions when taking account of socioeconomic circumstances and the institutional features of the education system. Additionally, only a handful of studies have examined how the influence of agency on early transitions might vary by socioeconomic background (e.g., Gil-Hernández, 2021). The present study stands out in examining the role of a forward-looking and situational dimension of individual agency in early transitions in the tracked system of Switzerland and in investigating how agency interacts with socioeconomic resources in shaping academic educational trajectories. Results indicate that both a forward-looking and a situational dimension of individual agency are important to embark on an academic path. However, a forward-looking orientation may be especially beneficial for students from socioeconomically advantaged origins, whereas exerting study effort seems to buffer the negative effect of scarce socioeconomic resources on the transition to academic tracks.

### Individual Agency and Socioeconomic Resources at Transitions

The first key contribution of this study is to assess the direct influences of individual agency and socioeconomic resources on early transitions. In line with the first hypothesis, the findings indicate that the two agency components investigated here—study effort and occupational aspirations—significantly predicted students’ probability of transitioning to academically demanding tracks. An adequate investment of children’s agentic resources seems to be required to successfully manage early transitions (Buchmann & Steinhoff, 2017). This is an important result because early educational transitions take place in a life stage that poses manifold developmental tasks (Buchmann & Kriesi, 2011). The exertion of agency at these early educational stages might not only affect later educational trajectories because of the opportunities provided by specific educational tracks. Agency in adolescence might also influence youth’s relationships with teachers, parents and peers, with important wide-ranging and long-lasting consequences on the life course (Wang & Fredricks, 2014). These results are in agreement with prior research on effort (Burger, 2021) and aspirations (Holtmann et al., 2021). By enriching the rather limited existing evidence, the findings corroborate the idea that to meet academic demands, students not only need to embrace a forward-looking orientation to motivate their behavior but also need to invest effort in the present (Schoon & Ng-Knight, 2017). As predicted by Hypothesis 2 and in line with existing evidence (e.g., Leemann et al., 2022), the results also indicate a positive link between family income and parental education, on the one hand, and the probability of transitioning to academic tracks at the lower- and upper-secondary education levels on the other hand. Thus, transitions from one educational level to another leave room not only for individual agency to play out but also for a wider constellation of socioeconomic resources to influence the transition.

### The Interplay between Individual Agency and Socioeconomic Resources at Transitions

The second key contribution of this study is to reveal how agency and family socioeconomic resources interact in shaping early educational transitions. This research found no evidence of a significant interplay between agentic and socioeconomic resources at the transition to lower-secondary education. However, the results suggest a significant interplay between agentic and socioeconomic resources in the transition to upper-secondary education, in agreement with Hypothesis 3 and with evidence from the United States on reading achievements (Liu, 2019). Specifically, the study found “resource multiplication” effects in the interplay between occupational aspirations and socioeconomic resources. Children from families with more socioeconomic resources may reap larger benefits from ambitious aspirations, eventually amplifying their overall advantage over less privileged children when coping with educational transitions. Relative to less socioeconomically privileged parents, parents from more advantaged backgrounds may better guide, encourage and support their children when navigating educational transitions via cultural and economic means and may, therefore, also be more able to support the attainment of their children’s aspirations. This result appears to be in line with evidence from Germany (Holtmann et al., 2021). However, it is at odds with findings from the United Kingdom which suggest that ambitious aspirations may be more beneficial to less advantaged groups, preventing absences from education, employment, or training (Schoon, 2014). These contradictory findings might be explained by the difference between Switzerland’s strongly tracked education system and the UK’s more comprehensive system. Educational tracks might in fact moderate the influences of both socioeconomic background and individual agency on educational transitions, and potentially of their interplay too (Burger, 2023).

By contrast, the study found “resource substitution” effects between study effort and socioeconomic resources. This suggests that children from less advantageous backgrounds, counting on fewer socioeconomic resources, may be relying more heavily on study effort to successfully make the transition to the academic track. An increased commitment to studying could function as a viable “substitute” for a lack of family socioeconomic resources, making the latter less consequential. This finding resonates with existing evidence (Schoon & Cook, 2021). When faced with an upcoming educational transition, exerting effort may constitute a tool for adolescents to compensate for a lack of socioeconomic resources available within the family.

Importantly, the study revealed very similar results for the interplay between children’s agency and the two separate dimensions of family socioeconomic resources (income and parental education). This is an important finding. Even though the financial and cultural assets available in the family might differentially benefit children’s educational outcomes (Pensiero & Schoon, 2019), the current findings suggest that income and parental education may moderate the influence of individual agency on educational transitions in very similar ways.

### Limitations and Recommendations for Future Research

Despite its strengths, this study is not without limitations. It was based on longitudinal observational data. As such, causal effects cannot be established unambiguously given potential omitted variable bias. Moreover, the measures of agency have some limitations. Self-reported items were used for occupational aspirations and study effort, which may open the way to social desirability bias, in particular with regard to study effort (Apascaritei et al., 2021). Replication of these findings using observational rather than self-reported measures, would therefore be warranted. Factor loadings for study effort were above the acceptable thresholds (Briggs & MacCallum, 2003) and should not represent a concern in this study (see results of a Monte Carlo simulation study by Ximénez, 2006). Nonetheless, some of them were relatively weak with potential implications for construct validity and the estimation of the structural parameters of interest. Sensitivity analyses indicated that results were robust against these potential threats. Replications of the analyses would be warranted to further test the robustness of the results when using different measurement models for study effort. Single-item assessments were used to measure aspirations, with potential implications for scale validity. Nonetheless, single-item assessments have long been used in large-scale surveys and generally indicate satisfactory face validity (Schoon et al., 2021). To handle missing data, this study used both full information maximum likelihood estimation and multiple imputation methods (see Online Resource S6 in the supplementary materials). The two procedures were implemented in comparable ways to ensure their equivalence in generating parameter estimates (Collins et al., 2001; see Online Resource S6 for a full discussion of the issue). Nonetheless, because minor discrepancies might arise between FIML-based and multiple imputation-based estimates, future studies should analyze the results obtained from the two estimators in more detail (Lee & Shi, 2021). Furthermore, for generalizability concerns, future research should conduct similar investigations in other tracked contexts, given the scarcity of evidence of this kind in tracked education systems. This study could not compare the relative importance of agentic and socioeconomic resources at the two transitions; this will require more targeted research designs and statistical tests. Furthermore, this study simultaneously investigated two dimensions of agency tapping into different temporal extensions, but it did not examine how aspirations may guide the effect of effort, essential to gaining an in-depth understanding of their function in influencing educational trajectories (Heckhausen & Buchmann, 2019). In a similar vein, future research should consider a broader range of dimensions of agency to unveil how different dimensions of agency shape educational attainment (Schoon & Heckhausen, 2019). Finally, this study did not investigate the role of parental co-agency (Buchmann et al., 2022), or of other actors (such as teachers) who are likely to help or hinder how students navigate early transitions in tracked systems (Buchmann et al., 2021).

## Conclusion

Early educational transitions in tracked education systems are decisive for children’s and adolescents’ subsequent educational trajectories. However, our understanding of the roles of individual agency and socioeconomic resources in early transitions is rather limited. This study extends existing research by investigating the independent influences of individual (present- and future-oriented) agency and socioeconomic resources, as well as their interplay, in early educational transitions in Switzerland. The study found that to successfully transition to academic tracks in secondary education, students not only rely on socioeconomic resources but also need to exhibit agency. Both an optimistic forward-looking orientation and the exertion of effort are important for students to make it to an academic track. Moreover, the results showed that the effect of agency on the transition to upper-secondary education depends on the socioeconomic resources available in the family. On the one hand, study effort emerged as a potentially valuable “substitutive” resource for less socioeconomically advantaged students. On the other hand, ambitious aspirations seem to be more beneficial for socioeconomically advantaged students. This latter evidence is concerning as it points to “hidden” mechanisms of social reproduction in education that may sediment very early on. Taken together, the evidence from this study contributes to our understanding of how early adolescents can pursue their educational trajectories within the boundaries imposed by socioeconomic circumstances and the structure of the education system. Importantly, the study calls for much greater attention to exploring not only the effect of individual agency on educational attainment but also its heterogeneity across different social groups.

### Supplementary information

Supplementary Information
