# Supplementary material for: Making it to the Academic Path in a Tracked Education System: The Interplay of Individual Agency and Social Origin in Early Educational Transitions
Source: J Youth Adolesc. 2023 Sep 2;52(12):2620–35. doi: 10.1007/s10964-023-01846-y (PMC10522750; doi:10.1007/s10964-023-01846-y)
Supplement: Supplementary file 1 — Supplementary Information [file 10964_2023_1846_MOESM1_ESM.docx]

**Online Supplementary Materials for:**

**Making it to the Academic Path in a Tracked Education System: The Interplay of Individual Agency and Social Origin** **in Early Educational Transitions**

**Online Resource S1: Zero-order Residual Correlations among Study Variables**

**Table S1** Zero-order correlations

|  | 1 | 2 | 3 | 4 | 5 | 6a | 6b | 6c | 7 |
| --- | --- | --- | --- | --- | --- | --- | --- | --- | --- |
| 1 Parental tertiary education | 1 |  |  |  |  |  |  |  |  |
| 2 Family income (log) | .444*** | 1 |  |  |  |  |  |  |  |
| Respondent at age 6 |  |  |  |  |  |  |  |  |  |
| 3 Male | .009 | -.034 | 1 |  |  |  |  |  |  |
| 4 Foreign language | -.118*** | -.134*** | -.042 | 1 |  |  |  |  |  |
| 5 Cognitive ability | .173*** | .178*** | -.059* | -.059* | 1 |  |  |  |  |
| Respondent at age 12 |  |  |  |  |  |  |  |  |  |
| Study Effort |  |  |  |  |  |  |  |  |  |
| 6a Apply myself to study/work | -.035 | -.026 | -.159*** | .129*** | .014 | 1 |  |  |  |
| 6b Try hard at school/work | .003 | .015 | -.123*** | .014 | -.010 | .433*** | 1 |  |  |
| 6c Do what is necessary for school/work | .098** | .102** | -.171*** | -.081* | .025 | .312*** | .220*** | 1 |  |
| 7 Occupational aspirations | .118*** | .163*** | .039 | .176*** | .057 | .076* | .077* | .156*** | 1 |
| 8 Early transition | .056 | .154*** | .090** | -.116*** | .110** | -.074* | -.115*** | -.003 | .034 |
| Respondent at age 14/15 |  |  |  |  |  |  |  |  |  |
| 9 Academic track – Lower-secondary | .268*** | .305*** | .0001 | -.068* | .177*** | .010 | .029 | .151*** | .222*** |
| 10 Long-term baccalaureate | .176*** | .150*** | -.051 | .019 | .078* | .014 | .122*** | .114*** | .082* |
| Study Effort |  |  |  |  |  |  |  |  |  |
| 11a Apply myself to study/work | -.039 | -.026 | -.173*** | .084* | -.013 | .396*** | .218*** | .196*** | .025 |
| 11b Try hard at school/work | -.038 | -.058 | -.153*** | .057 | -.023 | .258*** | .339*** | .120*** | -.088* |
| 11c Do what is necessary for school/work | -.031 | .015 | -.199*** | .031 | .006 | .301*** | .231*** | .338*** | .026 |
| 12 Occupational aspirations | .181* | .205*** | -.047 | .071* | .126*** | .069* | .109** | .138*** | .331*** |
| Respondent at age 16-18 |  |  |  |  |  |  |  |  |  |
| 13 Academic track – Upper-secondary | .333*** | .312*** | -.075* | .062 | .216*** | .098** | .088* | .172*** | .254*** |
| 14 Delayed transition | -.207*** | -.207*** | .097** | .041 | -.201*** | .002 | -.003 | -.121*** | -.203*** |
|  |  |  |  |  |  |  |  |  |  |

* *p* < .05, ** *p* < .01, *** *p* < .001

**Table S1** Zero-order correlations (continued)

|  | 8 | 9 | 10 | 11a | 11b | 11c | 12 | 13 | 14 |
| --- | --- | --- | --- | --- | --- | --- | --- | --- | --- |
| Respondent at age 14/15 |  |  |  |  |  |  |  |  |  |
| 9 Academic track – Lower-secondary | .133*** | 1 |  |  |  |  |  |  |  |
| 10 Long-term baccalaureate | -.029 | .257*** | 1 |  |  |  |  |  |  |
| Study Effort |  |  |  |  |  |  |  |  |  |
| 11a Apply myself to study/work | -.028 | -.009 | -.021 | 1 |  |  |  |  |  |
| 11b Try hard at school/work | -.162*** | -.052 | .005 | .547*** | 1 |  |  |  |  |
| 11c Do what is necessary for school/work | -.045 | .046 | .0004 | .549*** | .475*** | 1 |  |  |  |
| 12 Occupational aspirations | .056 | .302*** | .243*** | .075* | .05 | .105** | 1 |  |  |
| Respondent at age 16-18 |  |  |  |  |  |  |  |  |  |
| 13 Academic track – Upper-secondary | .118** | .403*** | .403*** | .063 | .031 | .101** | .471*** | 1 |  |
| 14 Delayed transition | .013 | -.260*** | -.178*** | .011 | .073* | -.013 | -.230*** | -.280*** | 1 |
|  |  |  |  |  |  |  |  |  |  |

* *p* < .05, ** *p* < .01, *** *p* < .001

**Online Resource S2: Longitudinal Measurement Invariance of Study Effort**

**Table S2** Tests for longitudinal measurement invariance for the construct of study effort

|  | χ^2^ | df | *p-value* | CFI | TLI | RMSEA | MC | Δ χ^2^ | Δ df | *p* | Δ CFI | BIC | AIC |
| --- | --- | --- | --- | --- | --- | --- | --- | --- | --- | --- | --- | --- | --- |
| *Study effort* |  |  |  |  |  |  |  |  |  |  |  |  |  |
| 1. Configural | 3.26 | 3 | .3533 | 1.000 | .999 | .009 |  |  |  |  |  | 15507.2 | 15388.3 |
| 1. Metric | 15.27 | 5 | .0093 | .992 | .977 | .044 | 2 vs 1 | 12.01 | 2 | .0025 | -.008 | 15505.3 | 15396.3 |
| 1. Scalar | 15.29 | 7 | .0324 | .994 | .987 | .034 | 3 vs 2 | .02 | 2 | .9878 | -.002 | 15491.4 | 15392.4 |

*Note.* df = degrees of freedom. CFI = comparative fit index. TLI = Tucker-Lewis index. RMSEA = root mean square error of approximation. MC = model comparison. Δ χ^2^ = χ^2^ difference. *Δ* CFI = change in CFI. *Δ* CFI and was computed by subtracting the CFI value of the more constrained model from the null-model (see model comparison). BIC = Bayesian information criterion. AIC = Akaike information criterion. The measurement models for study effort allow for the first two items to correlate consistently at each measurement point after scrutiny of modification indices that suggest such residual correlation at both measurement occasions. This, therefore, is likely to depend on the measurement structure rather than sample-specific variations (Little, 2013)

A set of confirmatory factor analyses (CFA) was performed to establish measurement invariance (MI) of the construct of study effort over time, to confirm that the same construct of study effort was measured across the two different time points (age of 12 and 15) (Cole & Maxwell, 2003). To establish MI, different measurement models were investigated, increasingly imposing different equality constraints on the constructs. The procedure began by identifying the measurement model. To set the scale of the constructs, the fixed factor scaling method was used, constraining the construct variance to 1 and the construct mean to 0 (as recommended by Little, Slegers, et al., 2006). Correlations across the two time points were specified between the construct variances and residual variances of correspondent items (Little, 2013).

Next, were investigated, in order, *configural* invariance (no equality constraints are imposed on the measurement model), *metric* invariance (equality constraints are added on correspondent factor loading at the two time points), and *scalar* invariance (equality constraints are also imposed on correspondent items’ intercepts at the two time points). As this study was interested in examining changes at the mean-level over time, measurement invariance at the *scalar* level needed to be established (Widaman et al., 2010). The differences between the three models with different equality constraints (metric vs. configural, and scalar vs. metric) were evaluated using the likelihood-ratio test. However, since the Δχ^2^ test is particularly sensitive to large sample size and negligible model misspecifications, the study also considered differences in comparative fit indices (Δ CFI), acceptable at a threshold of ≤ .01, which do not have these undesirable characteristics (Cheung & Rensvold, 2002). Table S2 displays the results of these analyses, showing that measurement invariance at the scalar level could be established for study effort across time.

**Online Resource S3: Procedure Used to Build Latent Interaction Terms for Study Effort**

Latent interaction terms were used to explore the moderation of the effect of study effort by parental education (Rhemtulla et al., 2012) and family income at the two transition points. The procedure that was used to build latent interaction terms followed the unconstrained product-indicator approach (Marsh et al., 2007), but the study used the residual centering approach to create the indicators loading on the latent interaction terms (see Little, Bovaird, et al., 2006). In total, four three-item latent interaction terms were created for each combination of the first-order variables of study effort (age 12), study effort (age 15), parental education, and family income. Taking the interactions between family income and study effort (age 12) as an example, the procedure was conducted as follows (in line with indications by Little et al., 2007; a graphical representation is displayed in Figure S1).

1. All possible product combinations of the corresponding indicators of the two first-order variables were used: three products were created between each item used for study effort (age 12) and family income.
2. Each of the three products was regressed onto all of the first-order manifest variables, i.e. all items used for study effort (age 12) and family income.
3. The three unstandardized residuals obtained from the three regressions were saved.
4. These residuals were then used as items’ loading on a latent interaction construct, which thereby defined the common variance among the three orthogonalized indicators.
5. All three items’ residuals of the latent interaction terms were allowed to correlate with each other.

The described approach yields multiple advantages. For example, the all-possible-combinations approach of the first-order manifest variables (as described in 1.), relative to other strategies for building product indicators (e.g., Marsh et al., 2007), ensures that all “the information about the interaction is fully captured by the indicators” of the latent interaction term (Little, 2013, p. 311). Furthermore, this approach enables full independence between the interaction term and first-order variables, interpretability of the main effects once the interaction is entered in the analysis, and unbiased and more stable estimates of the interaction term.


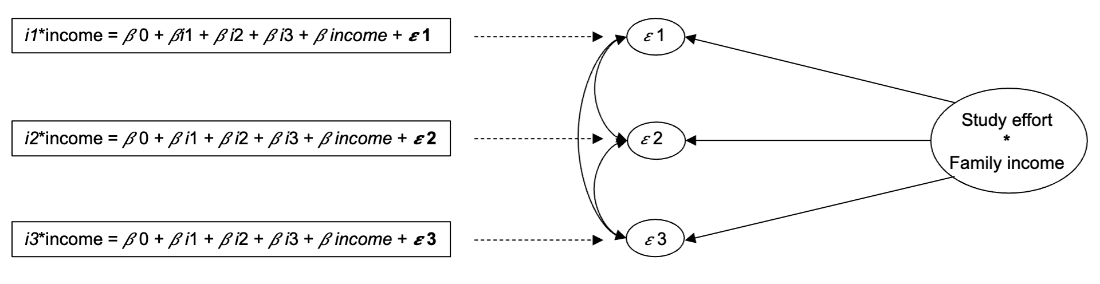


**Fig. S1** Graphical representation of the procedure to build the latent interaction terms. The interaction between study effort and family income is used as an explanatory example. “i1, i2, i3” refer to each of the items of study effort. **ε** refers to the unstandardized residual obtained from regressing the products between items of study effort and family income (e.g. i3*income)

**Online Resource S4: Figures of the Main Results from the Structural Equation Models 2 and 3**

**
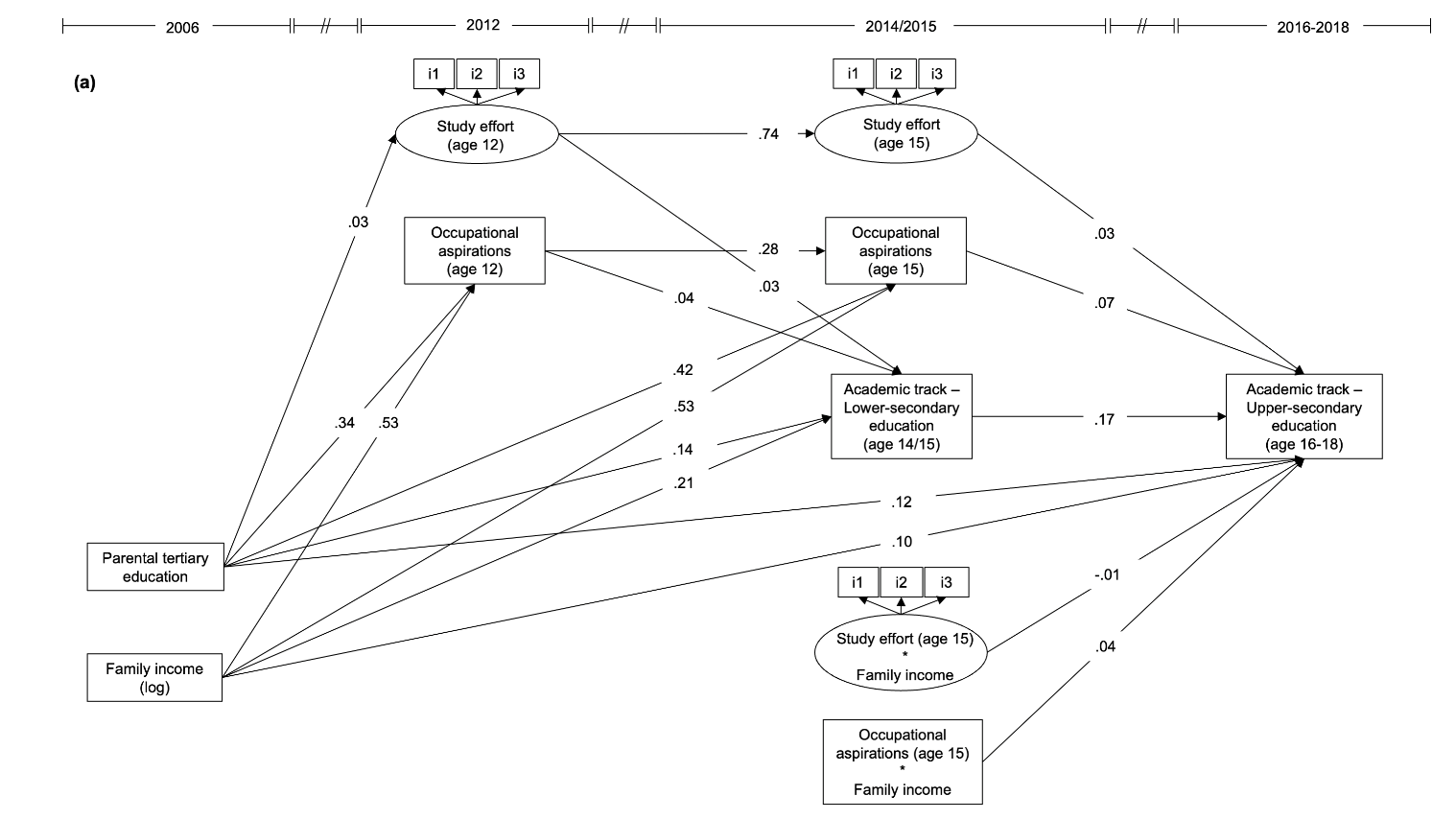
**

**
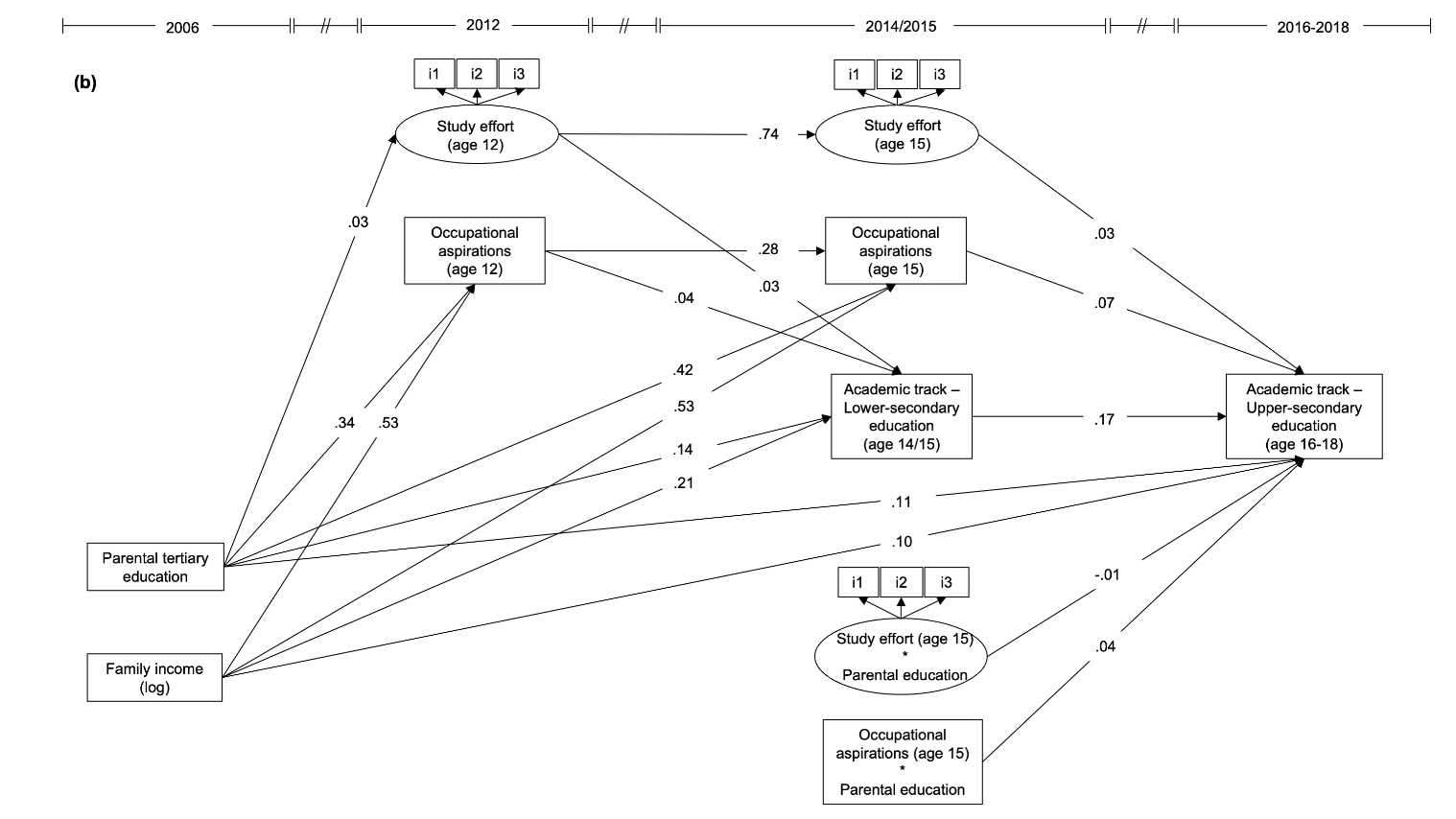
**

**Fig. S2** Structural equation model with interaction family income (top part (a) - Model 2) and parental education (bottom part (b) Model 3). Coefficients generated from linear probability structural equation models. Coefficients should be interpreted net the other effects estimated in the model. Unstandardized coefficients are reported, allowing interpretation of the results in their original metric. The remaining significant paths for the covariates—male, foreign language, cognitive ability, and controls for timing of the transitions—are not shown here for the sake of clarity (see Table 2)

**Online Resource S5: Path Coefficients from the Non-pruned Models**

**Table S3** Path coefficients from the non-pruned models

|  |  | Model 1.a | | Model 2.a | | Model 3.a | |
| --- | --- | --- | --- | --- | --- | --- | --- |
| Outcome | Predictor | Unstd.  Coefficient | SE | Unstd.  Coefficient | SE | Unstd.  Coefficient | SE |
| Occupational aspirations  (age 12) |  |  |  |  |  |  |  |
|  | Male | .178 | .195 | .178 | .196 | .178 | .195 |
|  | Parental tertiary education | .323*** | .025 | .323*** | .024 | .322*** | .022 |
|  | Family income (log) | .556*** | .018 | .557*** | .023 | .558*** | .023 |
|  | Foreign language | 1.147*** | .002 | 1.150*** | .009 | 1.149*** | .008 |
| Study effort  (age 12) |  |  |  |  |  |  |  |
|  | Male | -.619*** | .018 | -.619*** | .017 | -.619*** | .018 |
|  | Parental tertiary education | .051 | .148 | .051 | .148 | .051 | .150 |
|  | Family income (log) | .065 | .264 | .066 | .264 | .066 | .266 |
|  | Foreign language | .263 | .258 | .263 | .250 | .265 | .267 |
| Academic track –  Lower-secondary |  |  |  |  |  |  |  |
|  | Male | .025 | .016 | .025* | .010 | .025 | .014 |
|  | Parental tertiary education | .135*** | .029 | .135*** | .031 | .135*** | .029 |
|  | Family income (log) | .211*** | .013 | .211*** | .027 | .210*** | .016 |
|  | Foreign language | -.086*** | .019 | -.087*** | .020 | -.086*** | .017 |
|  | Cognitive ability | .028*** | .005 | .028*** | .005 | .028*** | .004 |
|  | Occupational aspirations (age 12) | .041*** | .0001 | .041*** | .002 | .041*** | .001 |
|  | Study effort (age 12) | .043* | .021 | .043* | .017 | .043 | .023 |
|  | Early transition | .083*** | .013 | .084*** | .002 | .083*** | .023 |
|  | Occupational aspirations (age 12) x Family income |  |  | -.008 | .071 |  |  |
|  | Study effort (age 12)  x Family income |  |  | -.001 | .002 |  |  |
|  | Occupational aspirations (age 12) x Parental education |  |  |  |  | -.003 | .042 |
|  | Study effort (age 12)  x Parental education |  |  |  |  | .006 | .004 |
| Occupational aspirations  (age 15) |  |  |  |  |  |  |  |
|  | Male | -.206 | .162 | -.205 | .163 | -.205 | .162 |
|  | Parental tertiary education | .416* | .208 | .416 | .213 | .415* | .206 |
|  | Family income (log) | .516 | .271 | .518 | .269 | .518 | .266 |
|  | Foreign language | .303*** | .047 | .308*** | .027 | .309*** | .055 |
|  | Occupational aspirations (age 12) | .285*** | .021 | .285*** | .022 | .285*** | .021 |
| Study effort  (age 15) |  |  |  |  |  |  |  |
|  | Male | -.216 | .166 | -.216 | .166 | -.216 | .167 |
|  | Parental tertiary education | -.173*** | .039 | -.174*** | .039 | -.174*** | .040 |
|  | Family income (log) | .024 | .046 | .024 | .045 | .024 | .046 |
|  | Foreign language | .170*** | .049 | .171*** | .049 | .169*** | .040 |
|  | Study effort (age 12) | .749*** | .033 | .749*** | .033 | .749*** | .033 |
| Academic track –  Upper-secondary |  |  |  |  |  |  |  |
|  | Male | -.020 | .013 | -.020* | .010 | -.021 | .011 |
|  | Parental tertiary education | .111*** | .026 | .114*** | .030 | .108*** | .014 |
|  | Family income (log) | .082** | .030 | .083*** | .005 | .086*** | .025 |
|  | Foreign language | .081** | .025 | .079*** | .024 | .074*** | .021 |
|  | Cognitive ability | .028*** | .007 | .027*** | .007 | .028*** | .007 |
|  | Early transition | .077 | .042 | .076 | .045 | .073 | .044 |
|  | Academic track –  Lower-secondary | .145* | .059 | .146** | .047 | .149** | .052 |
|  | Long-term baccalaureate | .403*** | .050 | .395*** | .041 | .395*** | .040 |
|  | Occupational aspirations (age 15) | .071** | .025 | .071*** | .020 | .071*** | .021 |
|  | Study effort (age 15) | .030*** | .005 | .030*** | .002 | .031*** | .002 |
|  | Delayed transition | -.091 | .060 | -.090 | .062 | -.090 | .061 |
|  | Occupational aspirations (age 15) x Family income |  |  | .040*** | .006 |  |  |
|  | Study effort (age 15)  x Family income |  |  | -.012*** | .003 |  |  |
|  | Occupational aspirations (age 15) x Parental education |  |  |  |  | .036** | .011 |
|  | Study effort (age 15)  x Parental education |  |  |  |  | -.014 | .008 |
|  |  |  |  |  |  |  |  |

*Note*. Unstd. = Unstandardized. SE = Cluster-robust standard errors. Unstandardized coefficients are useful for interpreting binary variables. Items for study effort were standardized before inclusion in the model (as in Schoon & Ng-Knight, 2017). Because of the clustering of standard errors by school tracks, the final sample used in the analysis counts 925 observations corresponding to the total number of observations in the different tracks. *df* = degrees of freedom. CFI = comparative fit index. TLI = Tucker-Lewis index. RMSEA = Root mean square error of approximation. CI = confidence interval

* *p* < .05, ** *p* < .01, *** *p* < .001

**Online Resource S6: Linear Probability Models**

To assist the interpretation of the interaction terms from the structural equation models (see Figure S2), predicted probabilities were also generated (see Figure 4 and Figure S4). Predicted probabilities were estimated using linear probability models (LPM), specified by tracing the rationale of the linear structural equation models. Estimating the linear probability models also allowed to further test the overall robustness of the results from the structural equation models. The probability of transitioning to academic tracks in upper-secondary school were regressed on all the variables used in the structural equation model (the study effort construct was introduced in the linear probability models using the factor scores predicted from the respective measurement models) (Model 4 below). The two interactions between occupational aspirations (age 15) and family income, and between study effort (age 15) and family income were entered in Model 5. The two interactions between the two agency constructs (age 15) and parental education were entered in Model 6.

**Table S4** Results from linear probability models

|  |  | Model 4 | Model 5 | Model 6 |
| --- | --- | --- | --- | --- |
| Outcome | Predictor | *β* | *β* | *β* |
| Academic track – Upper-secondary |  |  |  |  |
|  | Academic track – Lower-secondary | .137*** | .138*** | .139*** |
|  | Occupational aspirations (age 12) | .013 | .014 | .013 |
|  | Occupational aspirations (age 15) | .064*** | -.339* | .046*** |
|  | Study effort (age 12) | .043 | .040 | .042 |
|  | Study effort (age 15) | .034 | .416 | .047 |
|  | Male | -.026 | -.027 | -.026 |
|  | Parental tertiary education | .121*** | .122*** | -.068 |
|  | Family income (log) | .065* | -.122 | .068* |
|  | Foreign language | .064 | .065 | .062 |
|  | Cognitive ability | .027** | .027** | .026** |
|  | Early transition | .089** | .087** | .087** |
|  | Delayed transition | -.058 | -.058 | -.058 |
|  | Long-term baccalaureate | .394*** | .387*** | .387*** |
|  | Occupational aspirations (age 15) x Family income |  | .036** |  |
|  | Study effort (age 15) x Family income |  | -.034 |  |
|  | Occupational aspirations (age 15) x Parental education |  |  | .035** |
|  | Study effort (age 15) x Parental education |  |  | -.022 |
|  |  |  |  |  |

*Note.* N = 1,273*.* Linear probability coefficients (*β*) estimated on imputed data

* *p* < .05, ** *p* < .01, *** *p* < .001

To correct for potential bias related to the presence of missing data, this analysis used multiple imputation by chained equation (MICE). Multiple imputation replaces missing values with imputed data estimated from the observed data in the sample prior to the analysis. 100 datasets were generated (Graham et al., 2007) through a combination of linear regressions and predictive mean matching (*k*=10 as recommended by Morris et al., 2014). When missingness is related to observed variables incorporated in the analysis model (e.g., Enders, 2010)—as in the present case—multiple imputation generates more unbiased estimates of parameters compared to more traditional techniques such as listwise or pairwise deletion (Baraldi & Enders, 2010).

Multiple imputation was used instead of full information maximum likelihood estimation (FIML) which was used in the analyses reported in the main body of text. The two techniques generate equivalent results when 1) they use the same set of input data, 2) the number of imputations is sufficiently large, and 3) the imputation model is as complex as the analysis model in specifying the relationships among variables (Collins et al., 2001; Enders, 2010). The first two conditions are met in that FIML estimation and the multiple imputation model used the same set of input data, and the multiple imputation procedure applied a number of imputations considered acceptable to assume comparability with FIML-based estimates (Graham, 2012). As for the third condition, the imputation model used to estimate Models 5 and 6 (in Table S4) was more restrictive compared to the analysis models as it omitted the product terms between the two agency constructs and, family income and parental education. Models 5 and 6, and their correspondent predicted probabilities, were therefore replicated based on a further imputation model that included the interaction terms between the two agency constructs and, family income and parental education (see Models 5.a and 6.a in Table S5, respectively; the correspondent predicted probabilities are shown in Figure S3). Despite minor differences, the results from these additional analyses were consistent with the previous estimates of Models 5 and 6 and their correspondent predicted probabilities and led to the same conclusions.

**Table S5** Results from linear probability models based on adjusted imputation model including interaction terms

|  |  | Model 5.a | Model 6.a |
| --- | --- | --- | --- |
| Outcome | Predictor | *β* | *β* |
| Academic track – Upper-secondary |  |  |  |
|  | Academic track – Lower-secondary | .152*** | .153*** |
|  | Occupational aspirations (age 12) | .017* | .017* |
|  | Occupational aspirations (age 15) | -.288 | .034** |
|  | Study effort (age 12) | .044* | .046* |
|  | Study effort (age 15) | .517 | .051 |
|  | Male | -.027 | -.027 |
|  | Parental tertiary education | .127*** | -.043 |
|  | Family income (log) | .086 | .072* |
|  | Foreign language | .063 | .061 |
|  | Cognitive ability | .028** | .028** |
|  | Early transition | .084** | .084** |
|  | Delayed transition | -.070* | -.069* |
|  | Long-term baccalaureate | .396*** | .395*** |
|  | Occupational aspirations (age 15) x Family income | .030* |  |
|  | Study effort (age 15) x Family income | -.042 |  |
|  | Occupational aspirations (age 15) x Parental education |  | .032* |
|  | Study effort (age 15) x Parental education |  | -.026 |
|  |  |  |  |

*Note.* N = 1,273*.* Linear probability coefficients (*β*) estimated on imputed data (100 imputations) generated from an imputation model that included the interaction terms between the two agency constructs, and family income and parental education

* *p* < .05, ** *p* < .01, *** *p* < .001


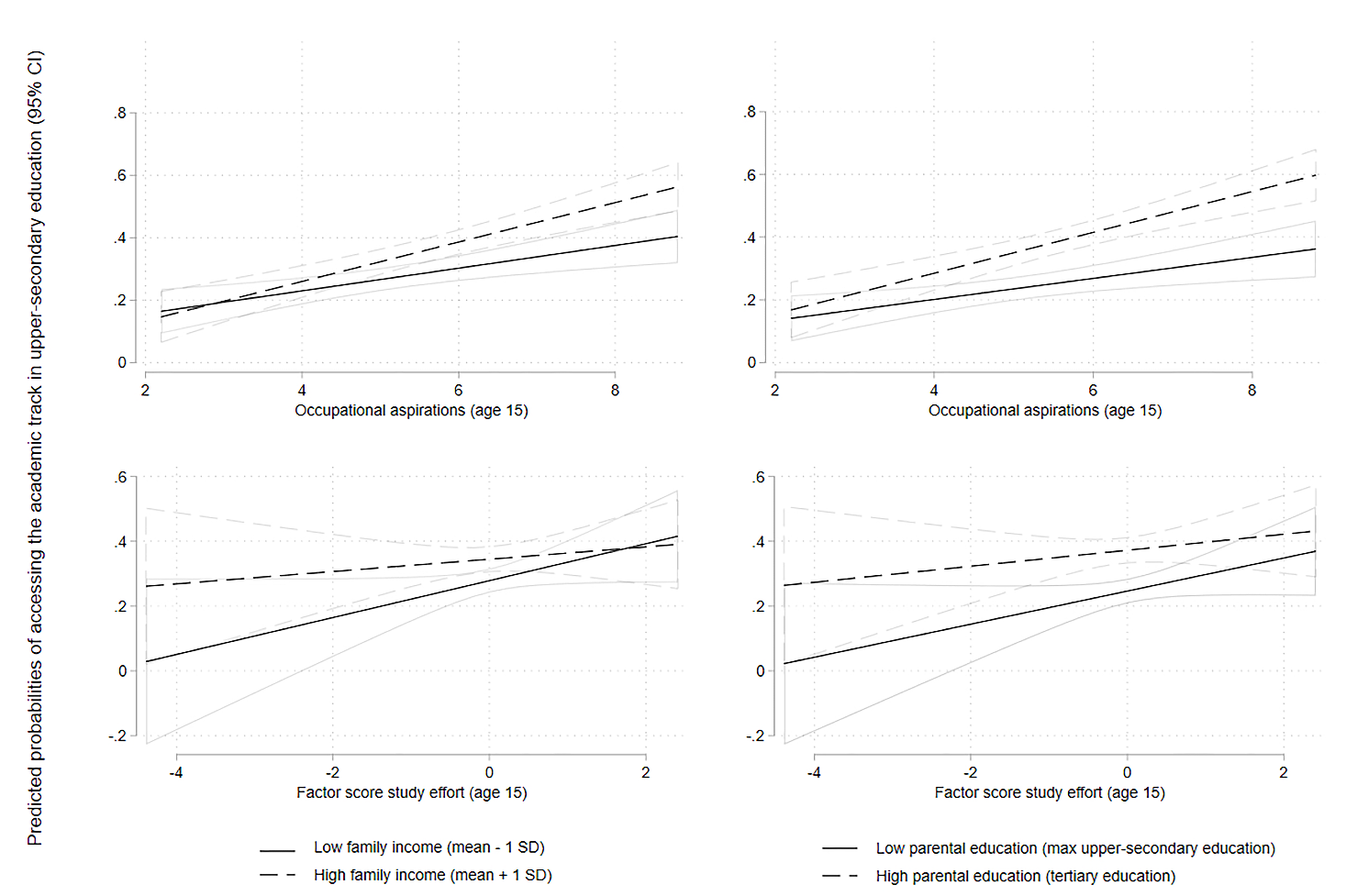


**Fig. S3** Predicted probabilities of transitioning to the academic track in upper-secondary education as a function of occupational aspirations (top row) and study effort (bottom row), at fixed values of family income (left column) and parental education (right column). Figures are based on Models 5.a and 6.a (in Table S5 above) estimated using an imputation model that included interaction terms between the two agency constructs, and family income and parental education. SD = Standard deviation

**Online Resource S7: Sensitivity Analysis**

**
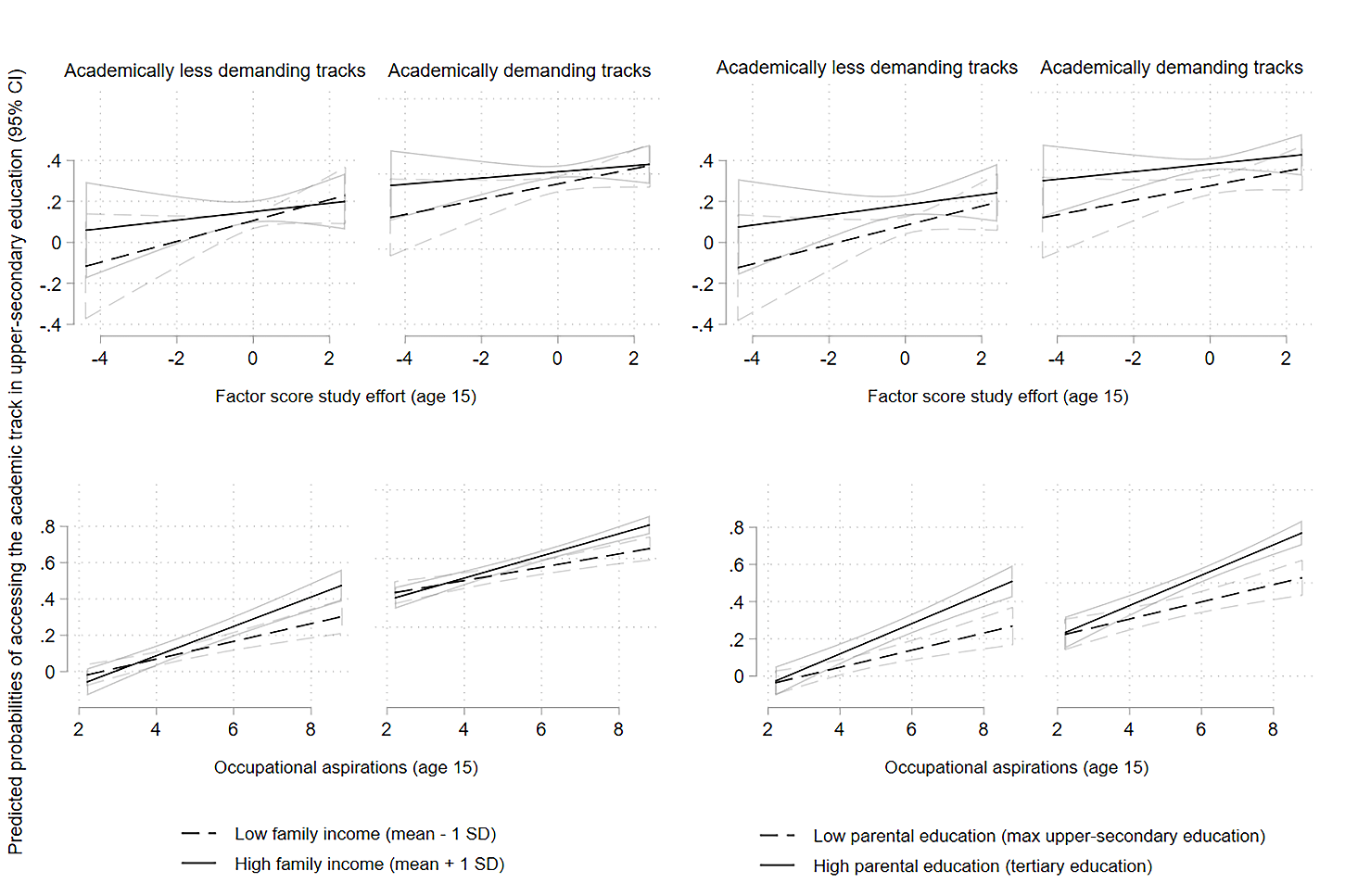
**

**Fig. S4** Predicted probabilities of transitioning to the academic track in upper-secondary education as a function of study effort (top row) and occupational aspirations (bottom row), at fixed values of family income (left column) and parental education (right column), by the track attended in lower-secondary education. SD = Standard deviation

**References**

Baraldi, A. N., & Enders, C. K. (2010). An introduction to modern missing data analyses. *Journal of School Psychology*, *48*(1), 5–37. https://doi.org/10.1016/j.jsp.2009.10.001

Cheung, G. W., & Rensvold, R. B. (2002). Evaluating goodness-of-fit indexes for testing measurement invariance. *Structural Equation Modeling: A Multidisciplinary Journal*, *9*(2), 233–255. https://doi.org/10.1207/S15328007SEM0902_5

Cole, D. A., & Maxwell, S. E. (2003). Testing mediational models with longitudinal data: Questions and tips in the use of structural equation modeling. *Journal of Abnormal Psychology*, *112*(4), 558–577. https://doi.org/10.1037/0021-843X.112.4.558

Collins, L. M., Schafer, J. L., & Kam, C.-M. (2001). A comparison of inclusive and restrictive strategies in modern missing data procedures. *Psychological Methods*, *6*(4), 330–351. https://doi.org/10.1037/1082-989X.6.4.330

Enders, C. K. (2010). *Applied missing data analysis*. New York: Guilford Press.

Graham, J. W. (2012). Analysis of missing data. In J. W. Graham (Ed.), *Missing data: Analysis and design* (pp. 47–69). New York: Springer. https://doi.org/10.1007/978-1-4614-4018-5_2

Graham, J. W., Olchowski, A. E., & Gilreath, T. D. (2007). How many imputations are really needed? Some practical clarifications of multiple imputation theory. *Prevention Science*, *8*(3), 206–213. https://doi.org/10.1007/s11121-007-0070-9

Little, T. D. (2013). *Longitudinal structural equation modeling*. New York: Guilford Press.

Little, T. D., Bovaird, J. A., & Widaman, K. F. (2006). On the merits of orthogonalizing powered and product terms: Implications for modeling interactions among latent variables. *Structural Equation Modeling: A Multidisciplinary Journal*, *13*(4), 497–519. https://doi.org/10.1207/s15328007sem1304_1

Little, T. D., Card, N. A., Bovaird, J. A., Preacher, K. J., & Crandall, C. S. (2007). Structural equation modeling of mediation and moderation with contextual factors. In T. D. Little, J. A. Bovaird, & N. A. Card (Eds.), *Modeling contextual effects in longitudinal studies* (pp. 207–230). Mahwah, NJ: Lawrence Erlbaum Associates.

Little, T. D., Slegers, D. W., & Card, N. A. (2006). A non-arbitrary method of identifying and scaling latent variables in SEM and MACS models. *Structural Equation Modeling: A Multidisciplinary Journal*, *13*(1), 59–72. https://doi.org/10.1207/s15328007sem1301_3

Marsh, H. W., Wen, Z., Hau, K.-T., Little, T. D., Bovaird, J. A., & Widaman, K. F. (2007). Unconstrained structural equation models of latent interactions: Contrasting residual- and mean-centered approaches. *Structural Equation Modeling: A Multidisciplinary Journal*, *14*(4), 570–580. https://doi.org/10.1080/10705510701303921

Morris, T. P., White, I. R., & Royston, P. (2014). Tuning multiple imputation by predictive mean matching and local residual draws. *BMC Medical Research Methodology*, *14*(1), 75. https://doi.org/10.1186/1471-2288-14-75

Rhemtulla, M., Brosseau-Liard, P. É., & Savalei, V. (2012). When can categorical variables be treated as continuous? A comparison of robust continuous and categorical SEM estimation methods under suboptimal conditions. *Psychological Methods*, *17*(3), 354–373. https://doi.org/10.1037/a0029315

Schoon, I., & Ng-Knight, T. (2017). Co-development of educational expectations and effort: Their antecedents and role as predictors of academic success. *Research in Human Development*, *14*(2), 161–176. https://doi.org/10.1080/15427609.2017.1305808

Widaman, K. F., Ferrer, E., & Conger, R. D. (2010). Factorial invariance within longitudinal structural equation models: Measuring the same construct across time. *Child Development Perspectives*, *4*(1), 10–18. https://doi.org/10.1111/j.1750-8606.2009.00110.x
